# The most common cow’s milk allergenic proteins regarding to allergic symptoms

**DOI:** 10.1186/2045-7022-1-S1-P62

**Published:** 2011-08-12

**Authors:** Raheleh Shokouhi Shoormasti, Zahra Pourpak, Zahra Yazdanyar, Zahra Lebaschi, Pegah Teymourpour, Saeedeh Barzegar, Behnaz Tazesh, Mohammed Reza Fazlollahi, Masoud Movahedi, Parisa Dashti, Mostafa Moin

**Affiliations:** 1 Immunology, Asthma and Allergy Research Institute, Tehran University of Medical Sciences, Tehran, Islamic Republic of Iran; 2 Department of Immunology and Allergy, Children Medical Center, Tehran University of Medical Sciences, Tehran, Islamic Republic of Iran

## Background

One of the most frequent food allergies in childhood is cow's milk allergy (CMA) that manifest with diverse symptoms. Cow's milk has four main proteins that their recognition can help to physician for patients treatment. The aim of this study was to determine the most common cow's milk allergenic proteins in children with CMA.

## Methods

All of children with the positive history of CMA and positive prick test &/or specific IgE were entered in this study. Specific IgE were determined for Cow's milk protein, α-lactalbumin, β-lactoglobulin, and casein and bovin serum albumin with RIDA Allergy Screen test. The specific IgE more than Class 2+ was acceptable.

## Results

Eighty six patients with CMA (3month to 18 yrs) were entered this study Fifty three patients were males (61.1%). Median of age was 2.5 yrs (Mean=3.98) and most of CMA patients was under 2 yrs (45.1 %). Mean of total IgE was 343.39±93.63 (SE)(0.2-5000IU/ml). The symptoms of patients was as following: (respiratory symptoms: 62.8%, Skin symptoms: 56.4%, gastrointestinal symptoms: 19.2% & Anaphylaxis: 13.6%). Totally, positive Specific IgE to cow's milk protein, α-lactalbumin, casein, β-lactoglobulin and bovin serum albumin were 89.2%, 78.3%, 75%, 63.4%, & 45.5%, respectively. Although α-lactalbumin & casein were the most allergenic protein, mean of specific IgE concentration to β-lactoglobulin was highest in patients with CMA. There was a significant correlation between positive specific IgE to β-lactalbumin & anaphylaxis (P=0.05) and also between respiratory symptoms and positive specific IgE to casein (P=0.01). There was significant difference between mean of specific IgE to casein between males and females (P=0.001) that was the more frequent in males.

## Conclusion

In this study, α-lactalbumin and casein seems to be the most allergenic protein in cow's milk but it seems that specific IgE positivity to β-lactoglobulin is related to anaphylaxis to CM.

